# Anti-miRs Competitively Inhibit microRNAs in Argonaute Complexes

**DOI:** 10.1371/journal.pone.0100951

**Published:** 2014-07-03

**Authors:** Daniel J. Hogan, Thomas M. Vincent, Sarah Fish, Eric G. Marcusson, Balkrishen Bhat, B. Nelson Chau, Dimitrios G. Zisoulis

**Affiliations:** Regulus Therapeutics, San Diego, California, United States of America; French National Center for Scientific Research - Institut de biologie moléculaire et cellulaire, France

## Abstract

MicroRNAs (miRNAs), small RNA molecules that post-transcriptionally regulate mRNA expression, are crucial in diverse developmental and physiological programs and their misregulation can lead to disease. Chemically modified oligonucleotides have been developed to modulate miRNA activity for therapeutic intervention in disease settings, but their mechanism of action has not been fully elucidated. Here we show that the miRNA inhibitors (anti-miRs) physically associate with Argonaute proteins in the context of the cognate target miRNA *in vitro* and *in vivo*. The association is mediated by the seed region of the miRNA and is sensitive to the placement of chemical modifications. Furthermore, the targeted miRNAs are stable and continue to be associated with Argonaute. Our results suggest that anti-miRs specifically associate with Argonaute-bound miRNAs, preventing association with target mRNAs, which leads to subsequent stabilization and thus increased expression of the targeted mRNAs.

## Introduction

miRNAs are a class of small (∼22 nt) genomically-encoded RNA molecules that inhibit translation initiation and stimulate decay of mRNA targets [1]–[3]. They are transcribed as long primary transcripts via RNA Polymerase II and cleaved by the RNAse III enzyme Drosha and its partner protein DGCR8 to form ∼70 nt hairpin loop structures (pre-miRNAs) [4]. Pre-miRNAs are exported to the cytoplasm where another RNAse III enzyme, Dicer, excises the loop resulting in the formation of a short double-stranded RNA molecule. One of the strands is loaded onto the Argonaute protein forming a ribonucleoprotein complex, the miRNA-mediated silencing complex (miRISC). miRNAs guide the complex to base-pair with imperfect complementarity to sequences in the target mRNAs, resulting in their subsequent destabilization and translational repression [1]–[4]. A key determinant of miRISC target sequence recognition mechanism is the “seed” region of the miRNA, nucleotides 2-8, that preferentially pair with sites on the regulated transcript [1], [5].

The hundreds of miRNAs encoded in mammalian genomes impact the translation rate and stability of most mRNAs [6] and are involved in diverse developmental and physiological programs, including metabolic and immune response pathways [7]–[10]. Mutation or misexpression of specific miRNAs is associated with a diverse array of malignancies, including prominent types of cancer [11]. It is possible to inhibit the function of specific miRNAs pharmacologically with complementary chemically modified oligonucleotides, called anti-miRs or antagomiRs [12]–[14]. Anti-miRs are complementary to the target miRNAs and contain chemical modifications to optimize the base-pairing affinity to the miRNAs, increase resistance to nucleases and add desirable pharmacokinetic properties [15]. anti-miRs are used to study loss of function effects of specific miRNAs and are being developed as therapeutics [13]. The first anti-miR to enter human clinical trials targets miR-122, an important host factor for Hepatitis C Virus infection and leads to multi-log reductions of the virus in patients [16]–[18]. A second clinical application is the inhibition of miR-21 in kidney fibrosis [19] and an anti-miR-21 compound is scheduled to enter human clinical trials in 2015.

Despite the therapeutic potential of anti-miRs, few studies have addressed their mechanism of action. In order to improve their pharmacological properties, such as potency, stability and distribution, anti-miRs are usually heavily chemically modified at positions in the backbone and on the sugar molecule [14]. However, the chemical modifications used may also affect the mechanisms by which they inhibit target miRNAs. First generation anti-miRs were complementary to full-length miRNA and included 2′-*O*-Methyl-modified bases with phosphorothioate groups near the 5′ and 3′ ends [20], [21]. These anti-miRs have poor pharmacokinetic properties and are thus not efficacious *in vivo* [21]. To facilitate tissue uptake they are often conjugated to a cholesterol moiety, which promotes accumulation in liver [21]. The seminal studies with 2′-*O*-Methyl anti-miRs suggested that they lead to destruction of the target miRNA, as evidenced by loss of signal via qPCR or Northern [21], [22]. However, subsequent studies with second generation anti-miRs containing 2′-Fluoro and 2′-Methoxyethyl groups (F/MOE), which improve favorable pharmacokinetic properties, showed that loss of signal of the targeted miRNA can be attributed to the anti-miR inhibiting detection of the miRNA due to the stability of the duplex [23]. More recently it was shown that 2′-*O*-Methyl anti-miRs induce 3′-end A-tailing and exonuclease trimming of the targeted miRNA [24]. RNA targets fully complimentary to the miRNA can induce ejection of the miRNA from Argonaute [25]; whether this phenomenon applies to anti-miRs is unknown. Since anti-miR chemical modifications in the studies mentioned above are different from those present in compounds in or nearing the clinic, which contain bicyclic sugar modifications, such as constrained Ethyl (cEt) and locked-nucleic acid (LNA), it is important to characterize the mechanism of action of anti-miR compounds with bicyclic sugar modification which hold therapeutic potential.

In this study we address several critical questions regarding anti-miR mode of action, utilizing a combination of biochemical assays and *in vivo* studies focusing on mouse liver. Our results suggest that anti-miRs primarily act through mature miRNAs engaged with Argonaute proteins via seed region pairing. Binding of the anti-miR to Argonaute prevents association with target mRNAs, leading to subsequent stabilization and thus increased expression of the targeted mRNAs. Subtle changes in the placement of bicyclic bases in anti-miRs can have dramatic effects on their ability to bind Argonaute-associated miRNAs, suggesting this may be an important determinant of efficacy.

## Materials and Methods

### Ethics Statement

Animal experiments in this work were limited to the harvest of tissues from humanely euthanized animals. The number of animals used was kept to the absolute minimum necessary to insure data quality (5 animals per group). The Regulus Therapeutics Inc. Institutional Animal Care and Use Committee approved all procedures. Briefly, mice were euthanized by exposure to isoflurane (5% v/v) until one minute after breathing stopped. Euthanasia was confirmed by cervical dislocation.

### Transgenic and wild-type Animals

*miR-21^-/-^* animals were generated as described previously [19]. Age and gender-matched C57BL6 wild-type animals used in these studies were purchased from Jackson Laboratories.

### Anti-miR administration

Age and gender-matched adult C57BL6 mice were administered anti-miR-21 or anti-miR-122 in Phosphate-Buffered Saline (PBS) solution by subcutaneous injection following the dosing regiment as described for each experiment in the Results section. Anti-let-7, anti-miR-21 and anti-miR-122 compounds are complementary to the 5′-end of let-7, miR-21 or miR-122, respectively, with a full phosphorothioate backbone and sugar modifications such as such as constrained Ethyl (cEt)/DNA or Fluoro/Methoxyethyl at the 2′ position of the sugar.

For cell culture experiments, anti-miRs were transfected using the Lipofectamine RNAiMax reagent (Life Technologies), at the indicated concentration, following the manufacturer's instructions.

### Immunopurification of Argonaute complexes and Northern Blot Analysis

Immunopurification of Argonaute from liver extracts and cultured cells was performed using the 4F9 antibody [26] essentially as described previously [3], [27], [28]. Briefly, 100–200 mg of fresh or flash-frozen liver samples were homogenized with a Dounce homogenizer in 2 ml of buffer B [20 mM Tris-HCl pH 8.0, 140 mM KCl, 5 mM EDTA pH 8.0, 0.5% NP-40, 0.1% deoxycholate, 100 U/ml Rnaseout (Life Technologies), 1 mM DTT and 1X Halt protease inhibitor cocktail (Pierce)]. The crude lysate was centrifuged at 1,000 g for 5 mins at 4°C. The supernatant was transferred to a new tube and centrifuged at 16,000 g for 5 mins at 4°C. The S16 supernatant was adjusted to 2 ml with buffer B and incubated with 10–20 ug of 4F9 antibody conjugated to epoxy magnetic beads (M-270 Dynalbeads, Life Technologies) for 2 hours at 4°C with gentle rotation. The beads were then collected by magnets, lysate was removed and the beads were washed three times five mins with 2 ml of buffer C [20 mM Tris-HCl pH 8.0, 140 mM KCl, 5 mM EDTA pH 8.0, 40 U/ml Rnaseout (Life Technologies), 1 mM DTT and 1X Halt protease inhibitor cocktail (Pierce)]. Following immunopurification, RNA and anti-miRs were extracted using standard phenol chloroform extraction methods. To isolate only the anti-miR compounds, RNA was hydrolyzed in the presence of 0.1N NaOH at 65°C for 15 mins, neutralized with 0.1 volume 1 M HEPES buffer. Oligonucleotides were precipitated with 0.1 volume sodium acetate and 5 volumes isopropanol at -20°C for 1 hr, centrifuged at 12,000 g for 15 mins at 4°C, washed with 70% ethanol.

anti-miRs were subjected to Northern blot analysis using Criterion 15% Tris-borate-EDTA (TBE)-Urea precast gels (Bio-Rad) following the manufacturer's recommendations. Oligonucleotides were transferred to N+ Nylon membrane (GE Healthcare), and UV-crosslinked following the manufacturer's protocol. The membrane was incubated with 3X saline-sodium citrate (SSC) solution with 0.1% sodium dodecyl sulphate (SDS). A Starfire (IDT DNA) probe for the sequence complementary to the anti-miR was prepared according to the manufacturer's instructions using [α-32P]-dATP and added to the hybridization solution for an overnight incubation at 42°C. The membrane was then washed three times in 2X SSC with 0.1% SDS, exposed to the phosphor imaging screen K (Bio-Rad) and developed using the Personal Molecular Imager FX Plus (Bio-Rad).

### Association of biotinylated compounds with Argonaute complexes and competition binding assays

Liver extracts were prepared as described above and the total protein concentration was adjusted to 10 mg/ml post centrifugation at 16,000 g for 15 mins at 4°C. Streptavidin-conjugated magnetic beads (MyOne T1, Life Technologies) were equilibrated with lysis buffer B and used to preclear the liver extracts for 30 mins at 4°C on a rocking mixer. The pre-cleared lysate was incubated with increasing amounts of 5′-biotinylated compounds for 2 hr at 4°C on a rocking mixer and washed three times for 5 mins with 1 ml of wash buffer. The samples were boiled in 1X LDS Sample buffer (Life Technologies) and loaded onto a pre-cast SDS-PAGE gel (Bio-Rad), transferred onto a PVDF membrane (Bio-Rad) and blocked using the manufacturer's instructions. Argonaute proteins were visualized using the mouse monoclonal anti-EIF2C2 antibody (2E12-1C9, Sigma-Aldrich) and a goat-anti-mouse secondary antibody conjugated to Horseradish Peroxidase (Jackson Immunoresearch labs), developed using the ECL Prime kit (GE Healthcare), digitized by the Fluorochem Q device (Cell Biosciences) and analyzed by the ImageJ software (NIH).

For the competition binding assay, Argonaute complexes were purified from liver or cell extracts using the 4F9 antibody or control IgA and immobilized on a 96-well electrochemiluminescence plate (Meso Scale Discovery), blocked, washed, incubated with an oligonucleotide complementary to the miRNA sequence (IDT DNA) and conjugated with a Sulfo-TAG (MSD) or a chemically modified oligonucleotide, following the manufacturer's instructions. Luminescence was quantified using the Sector Imager 2400 device (Meso Scale Discovery). Background levels were determined based on the luminescence levels of control IgA immunopurifications and were subtracted from the luminescence signal.

### RNA extraction and quantification of mRNAs

RNA from liver or cell line samples was extracted using the Qiagen RNeasy Kit or Qiagen miRNeasy Kit, following the manufacturer's instructions. mRNA levels were analyzed by qRT-PCR using Taqman One Step RT-PCR kit on a Viia 7 thermocycler (Life Technologies), relative to the GUSB housekeeping gene and processed using the ΔΔC_t_ method.

### Isolation and Quantification of miRNAs from anti-miR treated livers

Total RNA was isolated from 100 ul of S16 lysate (total RNA, post-16,000 g centrifugation) or 100 ul of resuspended Argonaute immunopurified samples by double extraction with Phenol:Chloroform:Isoamyl Alcohol (25∶24∶1, v/v) and one extraction with chloroform. 5 volumes of isopropanol, 1/10^th^ volume 3 M sodium acetate and 1 ul glycoblue (Life Technologies) were added to the extracted samples and RNA was precipitated by a 30 min centrifugation at 12,000 g at 4°C. The pellet was washed with 200 ul of ice-cold 70% ethanol, dried, and resuspended in 50 ul of RNAse-free water.

For the glyoxal treatment [29], 6 M glyoxal (Sigma) was deionized with 1 w/v AG 501-X8 resin (Bio-Rad) for 1 hour at RT on a rotator. Glyoxal working solutions were prepared with 5 ml deionized glyoxal, 6 ml DMSO and 10 mM sodium phosphate, pH 6.5. Aliquots were stored at −80°C. Equal volume of glyoxal working solution was added to RNA solutions and samples were incubated at 50°C for 30 min. Glyoxal was removed via miRNeasy kit (Qiagen) following manufacturor's protocol. Equal volume of reverse glyoxal solution (100 mM Tris-HCl, pH 8.0, 1 M KCl, 1 mM EDTA pH = 8.0) was added to purified RNA and samples were incubated overnight at 60°C. RNA was then purified by miRNeasy kit.

Nanostring hybridization was performed with nCounter mouse V1.2 miRNA expression assay (Nanostring) using 100 ng of total RNA or the equivalent miRNA content of 100 ng total RNA in Argonaute-immunopurified RNA, as measured by qRT-PCR of let-7A and miR-21.

### Melting curves

The melting temperatures for anti-miR-21-A and anti-miR-21-B oligonucleotide were determined as previously described [30]. The temperatures listed are an average of three experiments.

### Microarray Analysis

mRNA was profiled using Mouse Genome 430 2.0 array Human Genome 133 Plus v. 3 (Affymetrix) per manufacturer's instructions in triplicate. Cumulative distribution fractions were calculated as described previously [31].

## Results

### Anti-miRs associate with the Argonaute complex in vivo

**Figure 1:**
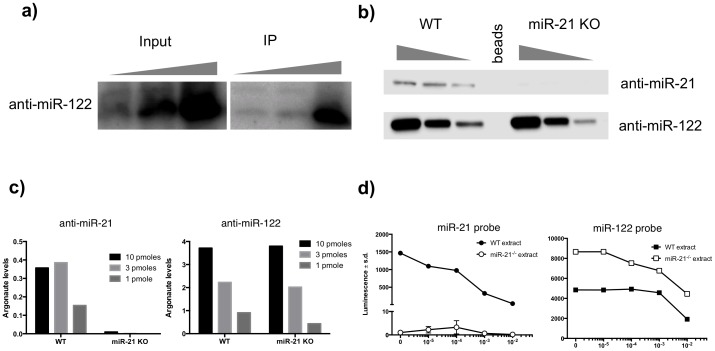
Anti-miRs specifically associate with the Argonaute *in vivo* and *in vitro*, only in the context of the cognate target miRNA. **a**) Northern analysis of anti-miR-122 in input lysate and in immunopurified Argonaute-containing complexes (IP) from liver tissue of animals dosed subcutaneously, three days prior, with 1, 3 or 10 mg/kg of the compound. Representative experiment shown from 2 independent experiments. **b**) Western analysis of the levels of Argonaute associated with biotinylated compounds in liver lysates from wild-type and *miR-21*-deficient animals. 10,3, or 1 pmoles of the 5′-biotinylated compounds were incubated with liver lysates, purified with streptavidin beads and the associated Argonaute proteins were visualized with an anti-Argonaute antibody. Representative experiment shown from three independent experiments. **c**) Quantification of Argonaute levels associated with the compounds from panel (**b**). **d**) Luminescence levels of a miR-21 probe bound by immunopurified miRNA:Argonaute complexes from wild-type or *miR-21*-lacking liver extracts (*miR-21^-/-^*), in the presence of increasing amounts of anti-miR-21. A miR-122 probe in the presence of increasing amounts of anti-miR-122 was used as a positive control for the assay. Representative experiments shown from 3 independent experiments (n = 3).

To gain insight into the mechanism of action of miRNA inhibitors, we sought to determine whether the anti-miRs physically associate with the Argonaute complex *in vivo*. To achieve this we performed Argonaute immunopurifications in liver lysates from anti-miR-122-treated mice and determined the levels of anti-miR associated with Argonaute (Figure 1a). Purified total RNA (and anti-miR) from input and Argonaute immunopurification samples were subjected to conditions that hydrolyze RNA without compromising the chemically modified anti-miRs, which were detected by Northern blot analysis. Anti-miRs can be detected in the input liver lysate, as expected, but also in the Argonaute immunopurifications, establishing that anti-miRs physically associate with the Argonaute complexes *in vivo*. Under typical immunoprecipitation conditions, we isolate approximately 30–40% of the Argonaute protein, and similar amounts of mature miRNAs (Figure S1) suggesting that the vast majority of mature miRNAs are associated with Argonaute and can be targeted by our anti-miRs.

### Anti-miRs associate specifically with the target miRNA in Argonaute

MiRNA inhibitors are designed to hybridize with the target miRNA sequence with high affinity [12], [13], [23]. To verify that the physical association of the anti-miR to the Argonaute complex is achieved only in the context of the target miRNA, we performed Argonaute/anti-miR binding assays in liver lysates from wild-type and *miR-21*-deficient mice [19] (Figure 1b). Increasing levels of the 5′-biotinylated anti-miR-21 were incubated with liver lysates from wild-type and *miR-21* knockout mice (*miR-21^-/-^*) and purified with streptavidin beads. The levels of Argonaute protein associated with the purified biotinylated oligo were visualized with Western blot analysis (Figure 1b,c). Argonaute proteins co-purify with biotinylated anti-miR-21 oligos in wild-type lysates but not in liver lysates lacking miR-21, suggesting that anti-miR-21 association with Argonaute is specifically mediated through the target miR-21. As a positive control, the biotinylated anti-miR-122 oligo associated with Argonaute in both wild-type and *miR-21*-deficient lysates. Interestingly, the Argonaute/anti-miR-21 association is saturated at 3 pmoles, while the Argonaute/anti-miR-122 association is not saturated (Figure 1b,c), reflecting the higher levels of miR-122 expressed in the liver lysate compared to miR-21.

We also verified our findings by employing a distinct, electrochemiluminescence-based competition binding assay (Figure 1d and S2). Briefly, Argonaute complexes were immunopurified from wild-type and *miR-21*-deficient liver lysate and incubated with a Ruthenium complex-labeled oligo complementary to the miR-21 sequence, in the presence of increasing amounts of the anti-miR-21 compound. Association of the compound with the miRNA:Argonaute complex in the wild-type lysate led to decrease in the Argonaute-associated luminescent probe as both probe and increasing amounts of compound compete for Argonaute association. In extracts lacking the target miRNA (*miR-21^-/-^* extract), the probe fails to associate with Argonaute, leading to background levels luminescence. Performing the experiment with a miR-122 probe and anti-miR-122 compounds yielded equivalent results for both lysate sources and served as a positive control for the assay. Taken together, our data suggest that anti-miRs associate with the miRNA:Argonaute complex only in the context of the targeted miRNA.

### Anti-miRs associate with the miRNA:Argonaute complexes via the seed region

**Figure 2:**
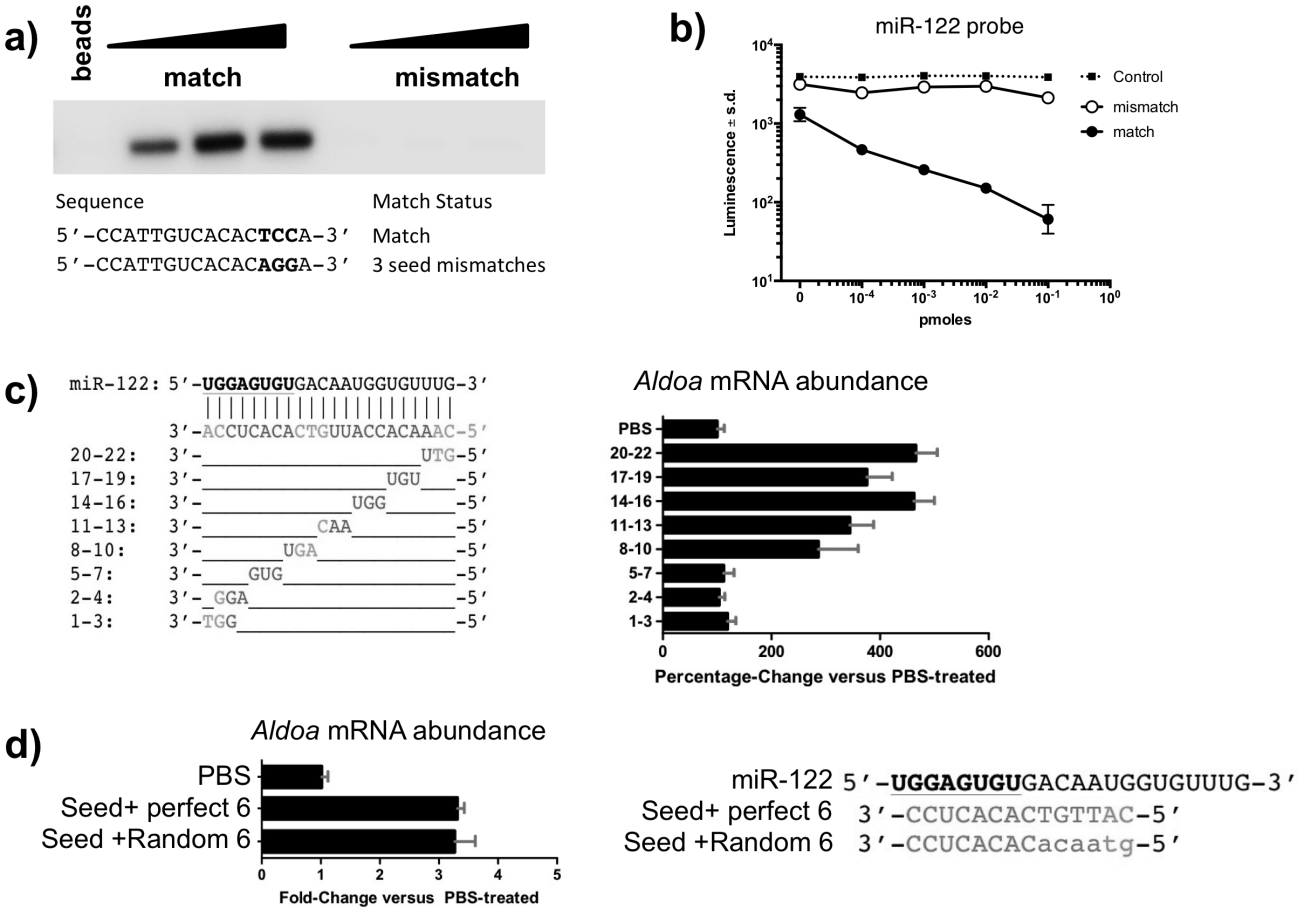
The seed region is an important determinant for anti-miRs to associate with the miRNA:Argonaute complexes *in vitro* and inhibit the target miRNA *in vivo*. **a**) Western analysis of the levels of Argonaute from liver lysates that associate with an anti-miR-122 compound with perfect seed matching (match) or with 3 mismatches in the seed region (mismatch). 10,3, or 1 pmoles of the 5′-biotinylated compounds were incubated with liver lysates, purified with streptavidin beads and the associated Argonaute proteins were visualized with an anti-Argonaute antibody. Representative experiment of 3 independent experiments. **b**) Luminescence levels of a miR-122 probe bound by Argonaute in the presence of an anti-miR-122 compound with perfect seed matching and its seed-mismatched counterpart (3 mismatches) or an unrelated negative control sequence. Match and mismatch sequences are as in (**a**). Representative experiment shown from 3 independent experiments (n = 3). **c**) mRNA levels of the miR-122-regulated *Aldoa* transcript in livers of mice treated with PBS or anti-miR-122 compounds with mismatches as shown in the table. Mice were treated subcutaneously for ten days with 25 mg/kg of the indicated compound. Results are shown as percentage of change over the PBS-treated animals ± s.d. Representative experiment from 2 independent experiments (n = 5). **d**) *Aldoa* mRNA levels in animals treated with anti-miR-122 compounds targeting the seed plus 6 complementary or random nucleotides. PBS controls or anti-miR solutions were administered subcutaneously at 25 mg/kg. Representative experiment from 2 independent experiments (n = 5).

The seed region of the miRNA, nucleotides 2-8, is a major determinant of miRNA targeting [1]. To investigate the importance of seed complementarity in the ability of anti-miRs to associate with the miRNA:Argonaute complex, we performed binding assays with 5′-biotinylated compounds with perfect and mismatched seed sequences (Figure 2). Not surprisingly, mismatches in the seed region abolished the binding ability of the anti-miR-122 for the Argonaute:miR-122 complex (Figure 2a) as evidenced by the inability of purified biotinylated mismatch compounds to co-purify with Argonaute. On the contrary, seed-matched compounds associated strongly with Argonaute complexes as visualized by Western blot analysis. We confirmed these results independently with the electrochemiluminescence-based competition-binding assay we developed (Figure 2b). As expected, the increasing concentrations of mismatched compound fail to compete with a miR-122-complementary probe, suggesting that the mismatch compound fails to associate with the miRNA:Argonaute complexes. However, the seed-matched version of the compound competed with the luminescent probe for association with Argonaute, as demonstrated by the reduced levels of Argonaute-bound luminescence with increasing concentrations of the compounds.

To further establish the importance of targeting the seed region of the miRNA in order to inhibit its function *in vivo*, we generated a series of anti-miR-122 with the same chemical modifications but with three mismatches in various locations along the anti-miR sequence (Figure 2c). The effect of anti-miRs on the derepression of *Aldoa*, a direct target of miR-122 [21], [32], was used to evaluate the ability of the compounds to inhibit miR-122 function. Mismatches in the compound sequence that corresponds to the target miRNA seed region, nucleotides 1–3, 2–4, 5–7 completely abolished the ability of the anti-miR-122 compound to inhibit miR-122 and derepress *Aldoa*, exhibiting approximately the same effect as injections of PBS solution. Mismatches in nucleotides 8–10 slightly diminished the potency of the anti-miR-122 compound, most likely due to the mismatched nucleotide 8, the last nucleotide in the seed region. However, mismatches in the non-seed region are well tolerated, yielding results equivalent to fully complementary miRNA inhibitors, typically a 4-fold derepression of the *Aldoa* target.

Since binding of the anti-miR to the target miRNA:Argonaute complex appears to be mediated by the miRNA seed, we investigated whether perfect complementarity with the target miRNA is required for inhibiting miRNA function. We generated two 14 nt-long anti-miR-122 compounds, encompassing the seed sequence plus 6 nucleotides, fully complementary or with a random sequence (Figure 2d), in order to control for pharmacokinetics properties *in vivo*. The ability of both compounds to derepress the miR-122 target *Aldoa* is similar (Figure 2d), suggesting that perfect complementarity outside the seed is not required for target engagement *in vivo*. Collectively, these results indicate that anti-miRs associate with the target miRNA:Argonaute complexes via base-pairing to the seed region of the target miRNA and there is no requirement for complementarity outside the seed sequence.

### The ability of anti-miRs to target miRNA:Argonaute complexes and inhibit their function in vitro and in vivo is affected by chemical modification patterns

**Figure 3:**
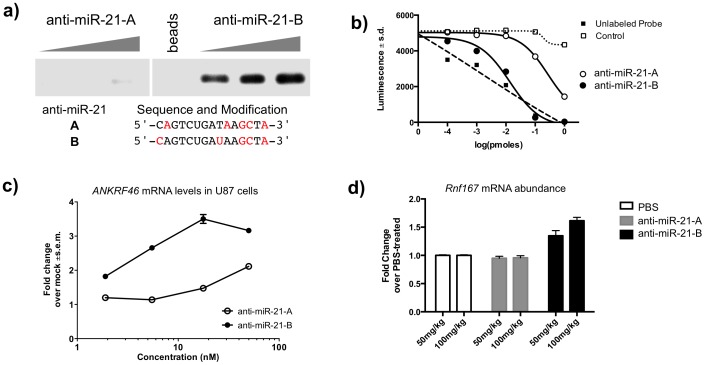
Subtle changes in the chemical modification pattern can affect anti-miR association with Argonaute and anti-miR activity *in vitro* and *in vivo*. **a**) Western analysis of the levels of Argonaute associated with anti-miR-21-A or anti-miR-21-B compounds, in liver lysates. These two compounds share the same sequence and chemistry, a phosphorothioate backbone with DNA and constrained Ethyl (red) bases. 10,3, or 1 pmoles of the 5′-biotinylated compounds were incubated with liver lysates, purified with streptavidin beads and the associated Argonaute proteins were visualized with an anti-Argonaute antibody. Samples from the same gel. Representative experiment from 3 independent experiments. **b**) Luminescence levels of a miR-21 probe bound by Argonaute in a competition binding assay with increasing amounts of anti-miR-21-A, anti-miR-21-B, unlabeled probe or an unrelated sequence as control. miRNA:Argonaute complexes were purified from HeLa cells. Representative experiment from 3 independent experiments (n = 3). **c**) mRNA levels of the miR-21-regulated *ANKRD46* gene in U87 cells transfected with increasing concentrations of anti-miR-21-A or anti-miR-21-B compounds. Results are shown as fold change over mock transfected ± s.e.m. Representative experiment from 2 independent experiments (n = 5). **d**) mRNA levels of the miR-21-regulated *Rnf167* transcript in livers of mice treated with PBS or the anti-miR-21 compounds A or B. Mice were injected subcutaneously once with 50 or 100 mg/kg of anti-miR-21-A or B, RNA was extracted from liver samples three days post-injection, and analyzed for *Rnf167* mRNA levels by qPCR. Results are shown as fold change over the PBS-treated group of animals ± s.d. Representative experiment from 2 independent experiments (n = 5).

A number of distinct chemical modifications can be incorporated into the design of an anti-miR to convey desired properties, such as improved stability, potency and pharmacokinetic properties [12], [15], [32]. To determine if the chemical modification pattern in anti-miRs can affect their ability to bind to their target miRNA in the Argonaute complex and thus influence their capacity to inhibit the miRNA:Argonaute complex *in vitro* and *in vivo*, we generated two anti-miR-21 compounds, of the same sequence but with slightly different chemical modification placement in 2 positions, termed compounds A and B, as shown in Figure 3a. Anti-miR-21-A and B contain a mix of constrained ethyl-modified (cEt) and DNA bases but differ in their placement at two specific locations as shown in the figure. For pairing to an adenosine in the target miRNA, we employ either a constrained ethyl uracil or a DNA thymidine. Both compounds have similar binding affinity to miR-21, as determined by the melting curve analysis with Tm = 72.7 and Tm = 72.2, respectively (Figure S3). We evaluated the ability of the compounds to associate with Argonaute *in vitro* (Figure 3a,b) and to derepress miR-21 targets in cell culture and *in vivo* (Figure 3c,d). Anti-miR-21-A barely co-purifies with Argonaute complexes from liver lysates, even at high levels of spiked 5′-biotinylated compound, while compound B can strongly associate with Argonaute at all levels (Figure 3a). According to the more sensitive electrochemiluminescence-based competition assay (Figure 3b), anti-miR-B is more potent at associating with the Argonaute-bound miR-21 than anti-miR A, as evidenced by the dramatic decrease in luminesce (anti-miR-A logIC_50_ = −0.42 pmoles, anti-miR-B logIC_50_ = −1.96 pmoles). The unrelated target sequence (let-7), was used as a negative control and did not compete with the probe. On the other hand increasing amounts of unlabeled probe competed with the labeled probe as expected, serving as a positive control.

Having established that subtle changes to the modification pattern can affect the ability of the anti-miR to target the Argonaute-bound miRNA, we sought to determine their effect on derepressing miR-21 mRNA targets in cell culture and *in vivo*. For this, we transfected U87 cells with increasing concentrations of anti-miR-21-A or anti-miR-21-B and examined their effect on the derepression of *ANKRD46* (Figure 3c), a target of miR-21 [33]. Treatment with anti-miR-B derepressed *ANKRD46* in a dose-response manner, increasing the mRNA levels ∼3.5 fold over mock-transfected. At the same concentration (17 nM), anti-miR-21-A barely increased *ANKRD46* mRNA levels. We also determined the effect of these compounds on the derepression of *Rnf167*, a miR-21 target [34], *in vivo* (Figure 3d). Anti-miR-21-B treatment led to a dose-response derepression of *Rnf167* of ∼1.6-fold at the high dose, while anti-miR-21-A response was at control levels. Taken together, these data indicate that alterations in the modification pattern of anti-miRs can affect their ability to target miRs in the Argonaute context and to inhibit the target miRNA:Argonaute function *in vitro* and *in vivo*.

### Anti-miR treatment leads to decreased association of Argonaute with target mRNAs increasing their abundance

**Figure 4:**
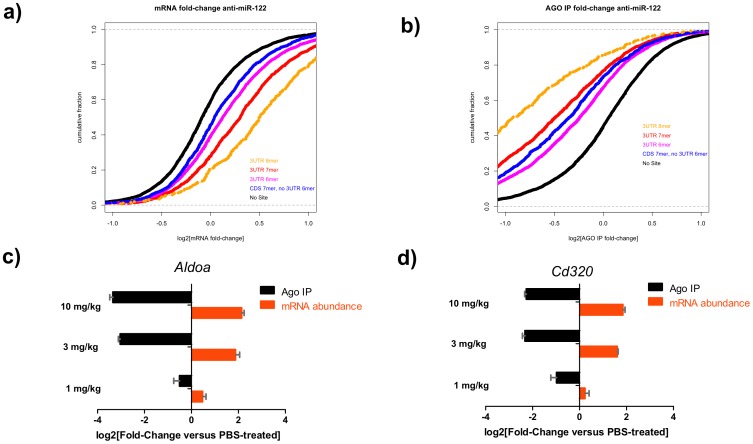
Anti-miR treatment decreases the levels of Argonaute-bound mRNA targets, increasing their stability and abundance *in vivo*. **a and b**) Cumulative-distribution fraction plots (CDF) depicting the mRNA fold change in total RNA (**a**) and in the Argonaute immunopurified (Ago) fraction (**b**) from liver lysates of animals dosed with with 10 1 mg/kg of anti-miR-122 as compared to PBS-treated controls as determined by microarray analysis. **c and d**) Total and Argonaute IP (Ago) RNA was assayed for the miR-122 targets (**c**) *Aldoa* and (**d**) *Cd320*. Mice were treated subcutaneously with 1, 3 or 10 mg/kg of an anti-miR-122 compound or PBS and livers were collected three days later. Results are displayed as fold-change±s.e.m.. Representative experiment from 2 independent experiments shown (n = 3).

Having established that anti-miRs associate with the target miRNA in the context of Argonaute we hypothesized that the anti-miR association with the miRNA:Argonaute complex prevents Argonaute-bound miRNAs from binding and regulating target mRNAs. We tested this hypothesis *in vivo* and *in vitro*, by performing Argonaute immunopurifications in liver lysates from mice treated with anti-miRs or from cell lines transfected with anti-miRs (Figure 4 and S4). We profiled total RNA and Argonaute-immunopurified fractions via microarray hybridization and focused on changes in mRNAs with with 6–7- or 8-nt seed matches as described previously [3], [31] (Figure 4a,b). mRNA transcripts with seed matches in the 3′UTR were derepressed in total mRNA compared to PBS-treated controls. Transcripts with 8-mer seed matches in the 3′UTR exhibited the highest derepression, followed by 7- and 6-mer matches, while seed-matches in the coding sequence (CDS) had the smallest effect (Figure 4a). In contrast, transcripts with 8-mer seed matches associated less with Argonaute in the presence of the anti-miR (Figure 4b). The levels of established miR-122 target genes *Aldoa* and *Cd320* [21], [35], [36] in the total lysate and in the Argonaute-associated fraction were also quantified in these samples by qPCR (Figure 4c,d). Treatment with the anti-miR-122 reduced the fraction of the targets associated with Argonaute compared to the PBS-treated samples, ∼6- and ∼4-fold respectively in a dose-dependent manner (Figure 4c,d). qPCR analysis of the relative abundance of *Aldoa* and *Cd320* mRNA transcripts compared to PBS-treated controls revealed a ∼4-fold increase for both transcripts.

We obtained similar results when we performed Argonaute immunopurification in HeLa cell lines transfected with anti-let-7 (Figure S4). The presence of the anti-miR led to a dramatic increase in the levels of the mRNA transcripts with 8-, 7- or 6-mer seed matches, while their association with Argonaute was dramatically reduced (Figure S4a,b). For the established let-7 targets *IGF2BP1* and *HMGA2* [37], [38] transcript abundance increased while Argonaute association was inhibited in a dose-response manner by the anti-let-7 treatment as determined by qPCR (Figure S4c). These results are consistent with the hypothesis stated above, that in the presence of an anti-miR, mRNA transcripts are no longer targeted and bound by the miRNA:Argonaute complexes which leads to an increase in their stability and thus their abundance; on the other hand, the miRNA:Argonaute complex is now occupied with the high-affinity anti-miR.

### Anti-miRs do not affect the levels of mature miRNAs or their association with Argonaute

**Figure 5:**
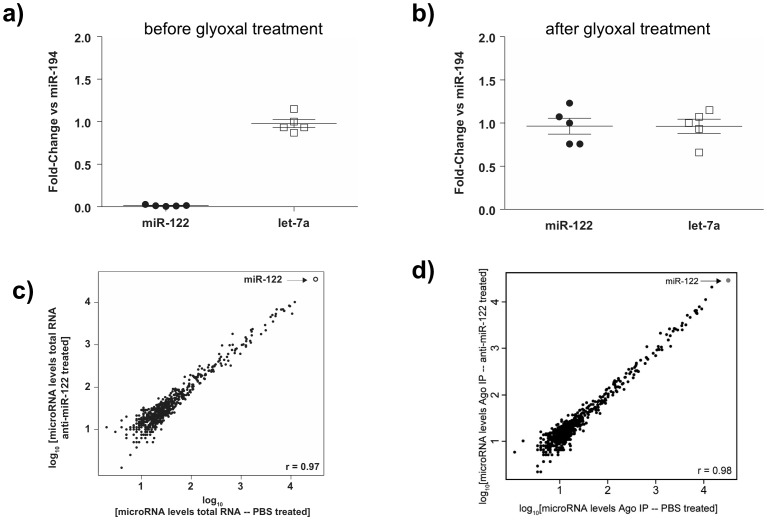
Anti-miRs do not affect the levels of total mature or Argonaute-bound miRNA *in vivo*. Total liver RNA isolated from animals 7 days post-subcutaneous administration of anti-miR-122 (10 mg/kg). miRNA levels in total RNA sample, profiled on the Nanostring platform, before (**a**) and after (**b**) the glyoxal treatment to separate the miR/anti-miR-122 duplex which interferes with miR-122 detection. **c and d**) Global miRNA levels, post-glyoxal treatment and profiled on the Nanostring platform, in anti-miR-122-treated animals compared to PBS-treated controls from total RNA (**c**) or immunopurified Argonaute fractions (Ago) (**d**). Representative experiments shown from 2 independent experiments (n = 3).

To investigate the effect of miRNA inhibitor treatment on the target miRNA and its association with Argonaute complex, we sought to determine if anti-miR treatment containing a mixture of DNA and constrained ethyl chemistry leads to degradation and/or ejection of the target miRNA from the Argonaute complex *in vivo* by performing Argonaute immunopurifications and RNA analysis (Figure 5). By design, anti-miRs form high-affinity duplexes with target miRNAs, a phenomenon that can interfere with miRNA detection obscuring the effect of anti-miR on the miRNA levels [23]. Although competitor-based methodologies have been developed to visualize the target miRNAs in the presence of ant-miRs [23], they are not compatible with quantitative and high-throughput miRNA level analysis. To address this issue, we subjected the immunopurified Argonaute-associated and total RNA fractions to glyoxal treatment [29], that allows the separation of anti-miR and target miRNA. Glyoxal is a dialdehyde that forms a reversible covalent adduct with guanosine, decreasing the affinity for the miRNA and enabling the separation of miRNAs and anti-miRs. This treatment allows for the selective purification of miRNAs using standard silica-membrane–based purification columns. Following this approach, we quantified the levels of miR-122 and let-7a miRNAs in the treated samples using qPCR before (Figure 5a) and after glyoxal treatment (Figure 5b) relative to miR-194 levels. Glyoxal treatment allowed for the detection of miR-122 in the treated samples at the same relative levels as let-7a, which was not targeted by an anti-miR. After establishing that we can accurately and quantitatively detect the levels of targeted miRNAs, we analyzed the global miRNA profile of the anti-miR-122-treated or PBS-treated samples, both for total RNA (Figure 5c and S5a) and immunopurified Argonaute-associated fractions (Figure 5d and S5b). The comparison between miR-122-treated *vs*. PBS-treated samples for both total RNA and Argonaute-associated fractions indicates that the relative levels of miR-122 remain mostly stable for both total lysate as well as in the Argonaute complex (Figure 5c,d and S5a,b). Taken together, these results suggest that treatment with a constrained ethyl/DNA anti-miR-122 does not affect the stability of mature miRNA levels or the miRNA:Argonaute association, in accordance with previously published results for anti-miRs with 2′-Fluoro and 2′-*O*-Methoxyethyl groups [23].

## Discussion

anti-miRs have emerged as a new class of molecular therapeutics for modulating master regulators of gene networks and as valuable tools to study the functions of specific miRNAs and identify their mRNA targets [13]. In the simplest case, a single-stranded oligonucleotide containing a phosphorothioate backbone and chemically modified bases can be injected subcutaneously and functionally accumulate into several tissues, including liver and kidney. Tissue uptake is mediated by binding to proteins in plasma via the phosphorothioate backbone [15]. Yet many of the details underlying anti-miR activity and their design principles are not fully understood. To gain insight into key mechanistic details of the anti-miR function, we investigated the requirements for their specific association with Argonaute-containing complexes and their effect on Argonaute-associated target miRNAs and miRNA-regulated mRNA transcripts. To our knowledge this is the first published study to provide direct evidence of the physical association of chemically modified oligonucleotides with the miRNA:Argonaute complex, both *in vivo* and *in vitro*. Based on a combination of biochemical and *in vivo* experiments on several anti-miRs we concluded that: (i) anti-miRs target the mature miRNA in the context of Argonaute, (ii) anti-miR specificity is largely conferred through pairing with the miRNA seed region, (iii) anti-miRs bind and inhibit the target miRNA, without significantly affecting miRNA stability, length, or Argonaute association and (iv) anti-miR association with the target miRNA:Argonaute complex increases the stability and abundance of target miRNA-regulated mRNAs.

We developed two methods to determine anti-miR association with miRNA:Argonaute complexes (Figures 1–3), allowing us to directly measure the relative affinities of different anti-miRs for their primary *in vivo* target. Our biochemical and *in vivo* studies support the importance of seed region pairing as reported previously [14], [39], and further show that subtle differences in placement of modified bases can have dramatic effects on the affinity for Argonaute-bound miRNAs. These assays can be adopted to screen libraries of anti-miRs to identify those with the highest affinity for their *in vivo* target either alone or in the context of a cell extract and to better characterize the relationship between the placement of chemical modifications and affinity for Argonaute-bound miRNAs.

Our observation that anti-miR-122 pairing to the miRNA seed region is necessary and in some cases sufficient for activity *in vivo* has several implications. Firstly, it suggests that mature miRNA is the primary target of anti-miRs; if effects on primary or precursor miR-122 were predominant, the seed region should not be more important than the rest of the mature miRNA. As a consequence, these results argue that anti-miRs specifically target mature miR-122 in complex with Argonaute; it is in the context of Argonaute that the miRNA seed region can strongly base-pair. The simplest explanation maybe that since the vast majority of miR-122, like most mature miRNAs, is Argonaute bound and its half-life is more than 10-fold longer than primary and pre-miR-122, there are greater chances for the anti-miR to interact with the mature miRNA in the context of Argonaute [40]–[42]. Nonetheless, it is possible that anti-miRs can target miRNA precursors and thus contribute to efficacy, as recently reported for a Locked Nucleic Acid (LNA) anti-miR-122 *in vitro* and in cell culture [43], yet it is unclear if this result can be attributed to the properties of the chemistry used.

The relative importance of the seed region for anti-miR activity can have serious implications on the principles of anti-miR design. Typically, high affinity bicyclic chemical modifications, such as LNA or constrained ethyl, are positioned across the anti-miR without significant emphasis on position. Our data suggest that anti-miRs could be thought of as bipartite with the region complementary to the seed dictating miRNA affinity, while the 5′-end of the anti-miR outside the seed could be used to promote favorable pharmacokinetic and safety properties. Many miRNAs exist as families, with a common seed region and some divergence elsewhere. Our results suggest that anti-miRs with high affinity seed region modifications designed against one member of a family will likely cross-react with other members, even with extensive mismatches outside of the seed region. Furthermore, since the seed sequence appears to be sufficient to mediate inhibition, shorter, mostly seed-containing anti-miRs could potentially minimize off-target interactions while retaining their activity against target miRNAs, as previously suggested [39].

Apart from desired pharmacokinetic properties and interactions with the target miRNA in the context of Argonaute [12], [13], [23], [32], chemical modifications seem to influence the anti-miR mechanism of action dictating the subsequent fate of the inhibited miRNA. Studies with 2′-*O*-Methyl anti-miRs suggested that they lead to destruction of the target miRNA, although later reports with anti-miRs containing 2′-Fluoro and 2′-*O*-Methoxyethyl groups, showed that loss of signal of the inhibited miRNA can be partly explained by the anti-miR interfering with the detection of the targeted miRNA [23]. A more recent report has shown that 2′-*O*-Methyl anti-miRs can induce trimming and tailing of the targeted miRNA [24]. In contrast, we did not find evidence for either phenomenon with the anti-miRs tested in this study (Figures 4-5), in agreement with previous studies employing 2′-Fluoro and 2′-*O*-Methoxyethyl modifications [23]. Why certain classes of anti-miR trigger trimming and tailing or induce Argonaute evacuation while others refrain from doing so is unclear, and it could be related to the structure of anti-miR and miRNA duplex in Argonaute. It may be possible to identify the requirements for such effects by testing a series of chimeric 2′-*O*-Methyl/constrained ethyl-DNA anti-miRs. Furthermore, anti-miRs with favorable *in vivo* properties conferred by bicyclic groups, that also induce destruction or ejection of the target miRNA may have favorable drug properties perhaps due to increased potency enabled by recycling of anti-miRs for multiple rounds of inhibition. Future experiments can explore the fate of targeted miR-122 and Argonaute, the half-lives of anti-miR, miRNA and Argonaute complexes *in vivo*, and if and how these complexes are turned-over or recycled.

**Figure 6:**
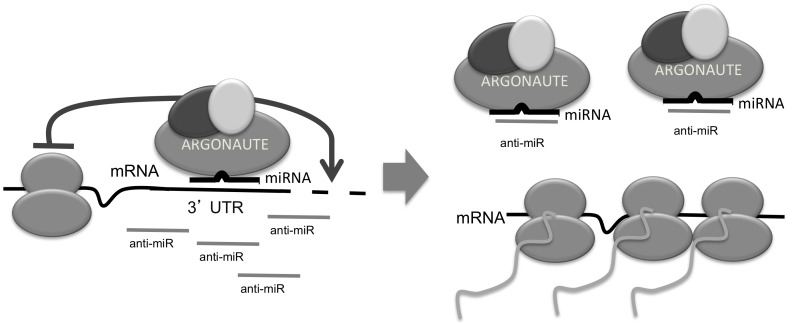
Model of the anti-miR mechanism of action. miRNAs, loaded onto Argonaute, guide the miRISC complex to target mRNA transcripts. The anti-miR specifically associates with Argonaute, in the context of the cognate target miRNA and now the miRNA:Argonaute complex can no longer bind and regulate target mRNAs. The mRNA targets are stabilized and can now be translated.

Concluding, we have shown that chemically modified oligonucleotides, designed to hybridize with high affinity to target miRNAs, associate with Argonaute complexes only in the presence of the cognate target miRNA. The association is guided primarily by hybridization to the seed region of the miRNA and is sensitive to the placement of high-affinity chemical modifications of the nucleotide bases. The targeted miRNA is mostly stable and continues to be associated with the Argonaute-containing complex. Our data are consistent with a model where competition between the target mRNA and the anti-miR compound for miRNA:Argonaute complexes leads to increased levels of stabilized mRNA targets (Figure 6).

## Supporting Information

Figure S1 **Most miRNAs in HeLa cells and mouse liver are associated with Argonaute.** **a**) Western analysis of Argonaute immunopurifications from HeLa cell lysate. **b**) Barplot showing the percentage of each species immunopurified with Argonaute. Let-7a, miR-16, miR-21 and U6 were measured by qPCR. The error bars represent the standard error of the mean from five replicates. **c**) Scatterplot between Nanostring hybridization signal measured from HeLa lysate (x-axis) and Argonaute immunopurifications (y-axis) for ∼200 miRNAs. **d**) Western analysis of Argonaute immunopurifications from mouse liver lysate. **e**) Barplot showing the percentage of each species immunopurified with Argonaute from mouse liver lysates. miR-122, miR-21 and U6 were measured by qPCR. The error bars represent the standard error of the mean from four replicates.
(TIFF)

Figure S2 **Schematic diagram of the competition binding assay.** An electrochemiluminescence plate is coated with the anti-Argonaute (4F9) or isotype control antibody (IgA). Liver or cell lysate is added to the well and following incubation, the Argonaute complexes are purified and immobilized on the plate while unbound lysate proteins are removed by washing. Increasing amounts of an anti-miR are added to the wells, as well as a constant amount of the probe, which is a modified oligonucleotide that can bind to the miRNA of choice, conjugated to a Ruthenium complex (S-TAG). Following incubation, unbound probe and anti-miR are washed away, and the probe signal is quantified with the addition of the detection reagent on a Mesoscale device. Background levels are determined based on the luminescence levels of control IgA immunopurifications and were subtracted from the luminescence signal.
(TIFF)

Figure S3 **Melting temperature curves of anti-miR-21-A and anti-miR-21-B by UV analysis.** Absorption at 260 nm of miR-21 and compound anti-miR-21-A or anti-miR-21-B, from 15°C to 95°C. Average absorption of 3 measurements.
(TIFF)

Figure S4 **Anti-miR treatment decreases the levels of Argonaute bound mRNA targets, increasing their stability and abundance** ***in vitro*** **.** **a and b**) Cumulative-distribution fraction plots (CDF) depicting the mRNA fold change in total RNA (**a**) and in the Argonaute immunopurified fraction (**b**) in HeLa cells treated with anti-let-7 (10 nM) compared to mock-treated cells as determined by microrarray analysis. **c**) Cell lysates from HeLa cells transfected with anti-let-7 compounds and were subjected to Argonaute immunopurifications, RNA was extracted and mRNA levels of the let-7 targets IGF2BP1 and HMGA2 were assayed by qPCR and represented as fold-change±s.e.m.. Representative experiment shown (n = 2).
(TIFF)

Figure S5 **AntimiRs do not affect the levels of total mature or Argonaute-bound miR-122** ***in vivo*** **.** **a**) Histogram of the fold-changes for ∼200 miRNAs comparing anti-miR-122 treated livers vs PBS-treated livers following glyoxal removal of anti-miR. The log2 values across all miRNAs were mean centered at zero. miR-122 fold-change is highlighted by the red line (log2 = 0). **b**) Same as in (**a**) except for Argonaute IP RNA: miR-122 fold-change log2 = 0.77. Representative experiments shown from 2 independent experiments (n = 3).
(TIFF)
