# Supplementary figures and images for: Anti-miRs Competitively Inhibit microRNAs in Argonaute Complexes
Source: PLoS One. 2014 Jul 3;9(7):e100951. doi: 10.1371/journal.pone.0100951 (PMC4084633; doi:10.1371/journal.pone.0100951)

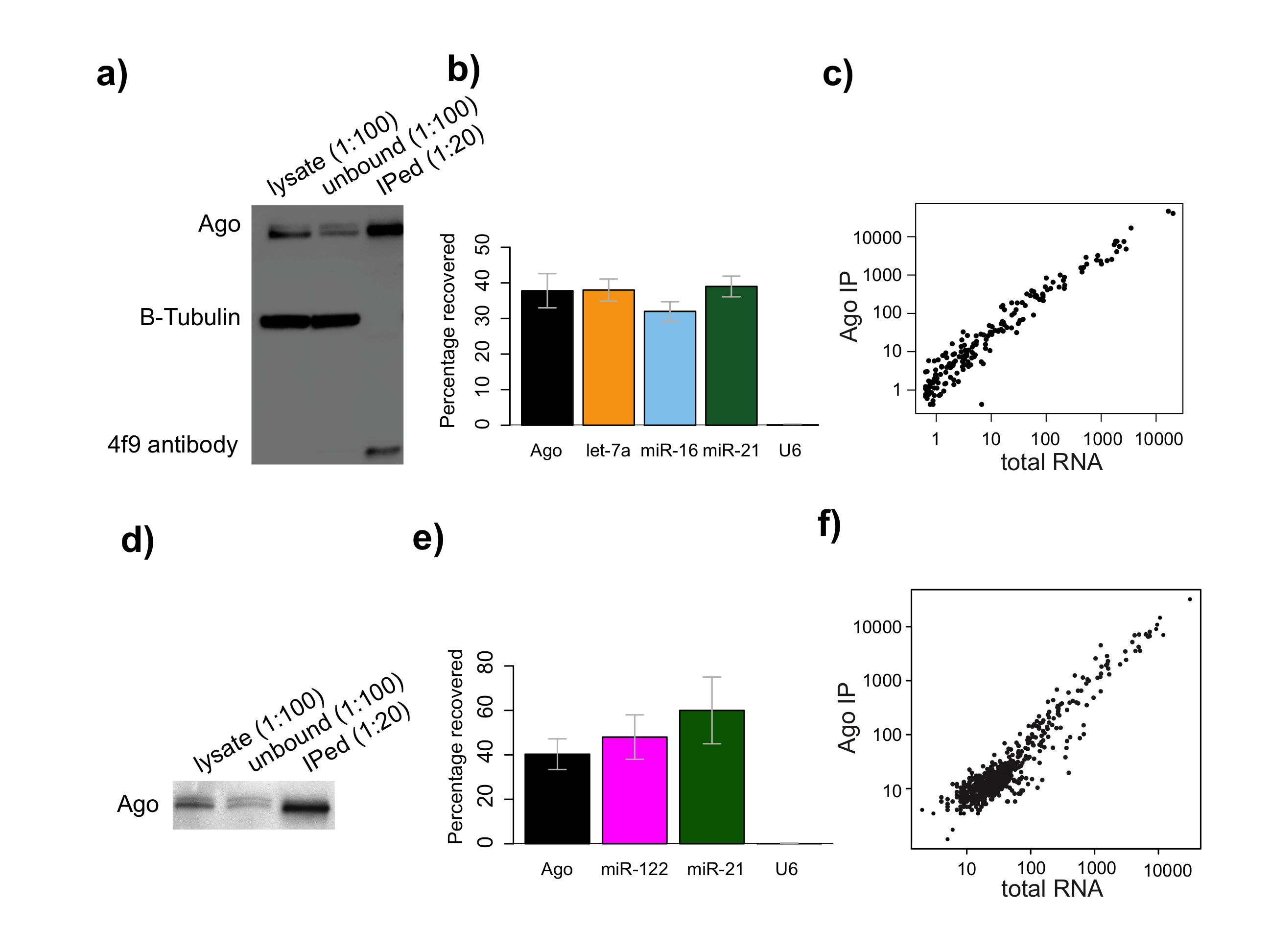

Supplement: Figure S1 — Most miRNAs in HeLa cells and mouse liver are associated with Argonaute. a) Western analysis of Argonaute immunopurifications from HeLa cell lysate. b) Barplot showing the percentage of each species immunopurified with Argonaute. Let-7a, miR-16, miR-21 and U6 were measured by qPCR. The error bars represent the standard error of the mean from five replicates. c) Scatterplot between Nanostring hybridization signal measured from HeLa lysate (x-axis) and Argonaute immunopurifications (y-axis) for ∼200 miRNAs. d) Western analysis of Argonaute immunopurifications from mouse liver lysate. e) Barplot showing the percentage of each species immunopurified with Argonaute from mouse liver lysates. miR-122, miR-21 and U6 were measured by qPCR. The error bars represent the standard error of the mean from four replicates. (TIFF) [file pone.0100951.s001.tiff]

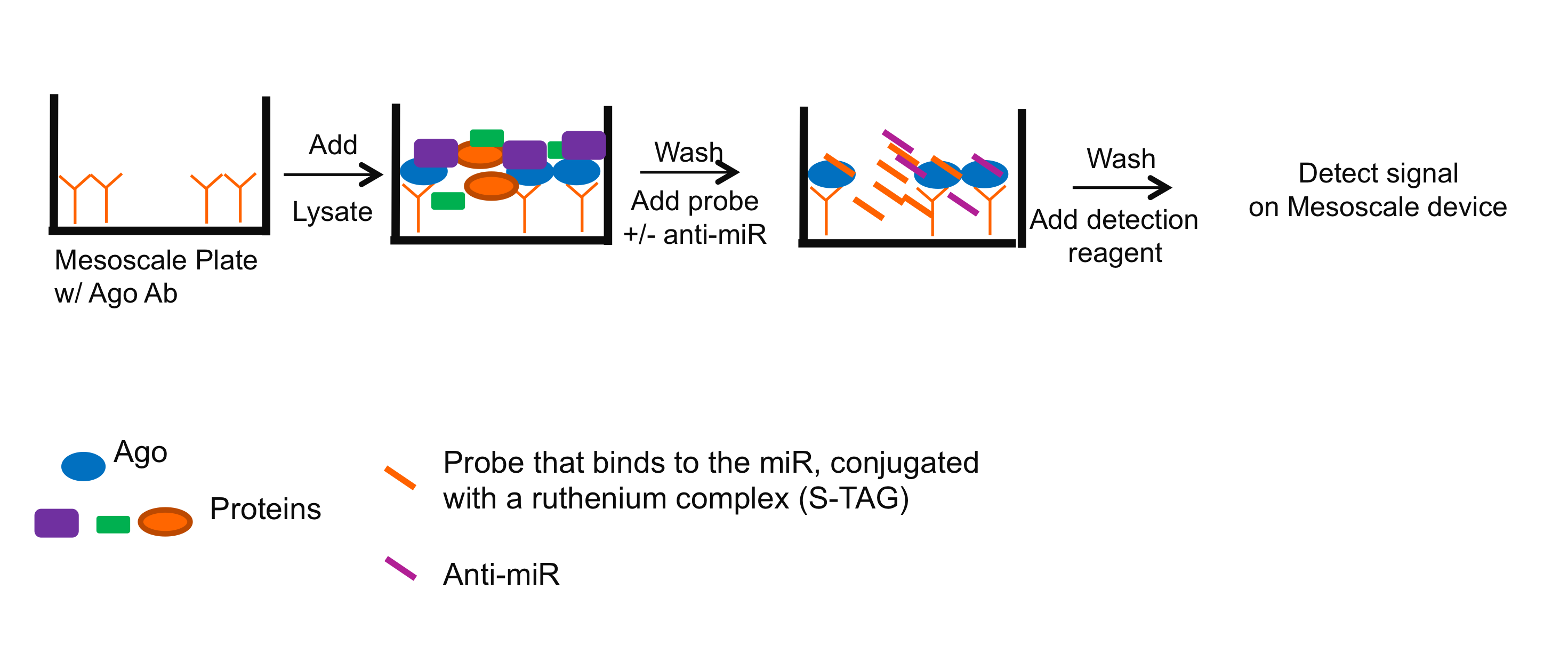

Supplement: Figure S2 — Schematic diagram of the competition binding assay. An electrochemiluminescence plate is coated with the anti-Argonaute (4F9) or isotype control antibody (IgA). Liver or cell lysate is added to the well and following incubation, the Argonaute complexes are purified and immobilized on the plate while unbound lysate proteins are removed by washing. Increasing amounts of an anti-miR are added to the wells, as well as a constant amount of the probe, which is a modified oligonucleotide that can bind to the miRNA of choice, conjugated to a Ruthenium complex (S-TAG). Following incubation, unbound probe and anti-miR are washed away, and the probe signal is quantified with the addition of the detection reagent on a Mesoscale device. Background levels are determined based on the luminescence levels of control IgA immunopurifications and were subtracted from the luminescence signal. (TIFF) [file pone.0100951.s002.tiff]

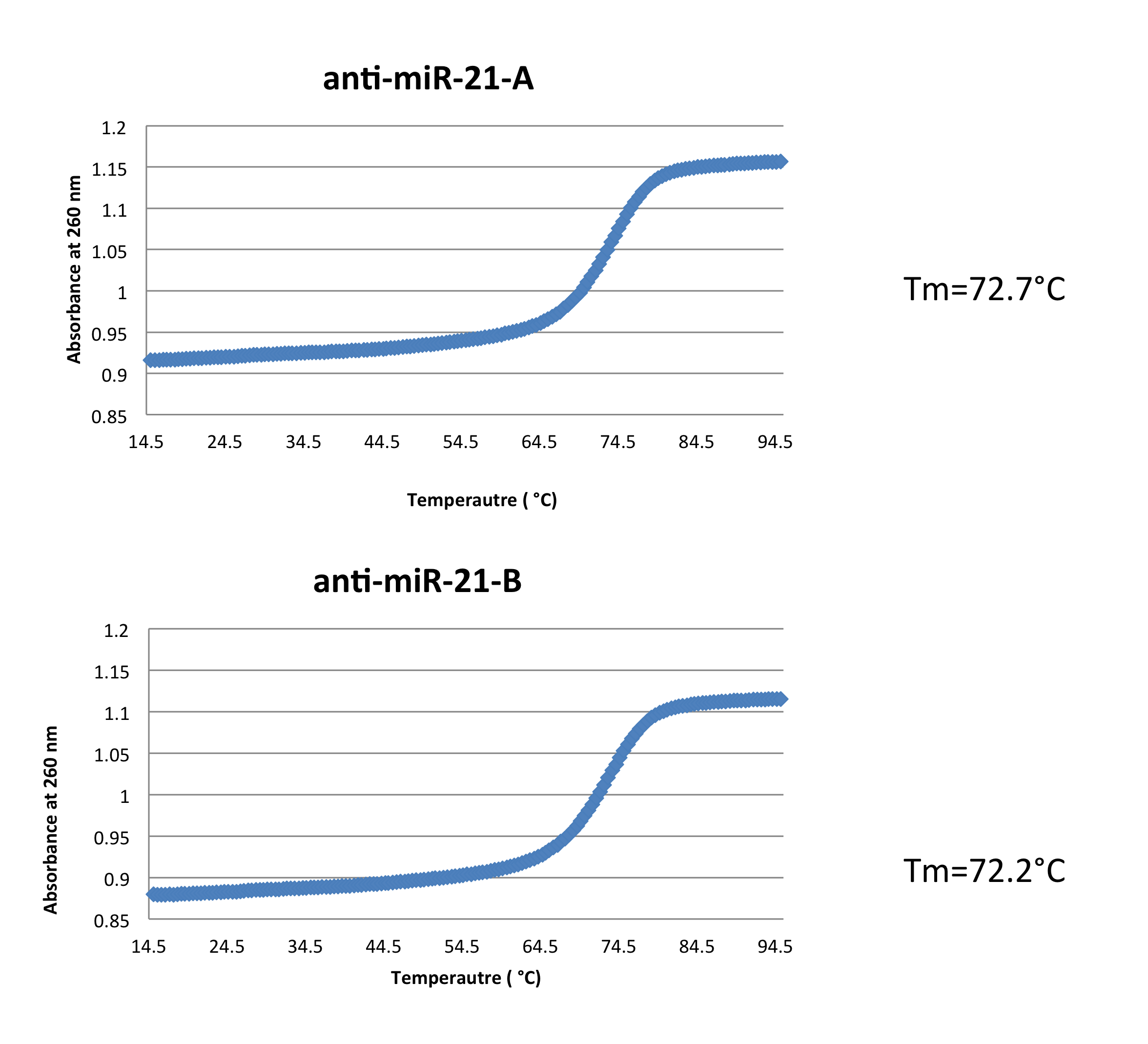

Supplement: Figure S3 — Melting temperature curves of anti-miR-21-A and anti-miR-21-B by UV analysis. Absorption at 260 nm of miR-21 and compound anti-miR-21-A or anti-miR-21-B, from 15°C to 95°C. Average absorption of 3 measurements. (TIFF) [file pone.0100951.s003.tiff]

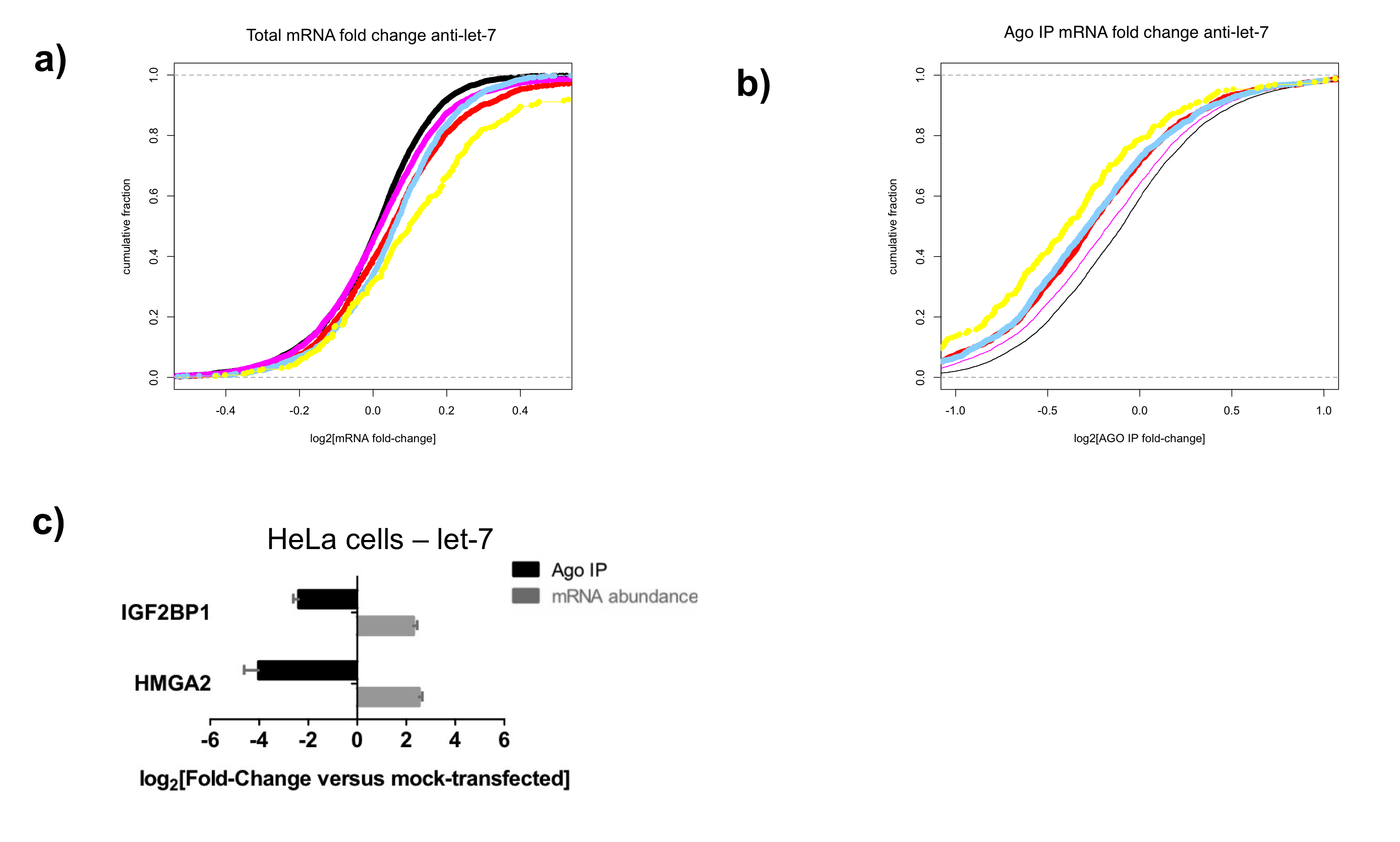

Supplement: Figure S4 — Anti-miR treatment decreases the levels of Argonaute bound mRNA targets, increasing their stability and abundance in vitro . a and b) Cumulative-distribution fraction plots (CDF) depicting the mRNA fold change in total RNA (a) and in the Argonaute immunopurified fraction (b) in HeLa cells treated with anti-let-7 (10 nM) compared to mock-treated cells as determined by microrarray analysis. c) Cell lysates from HeLa cells transfected with anti-let-7 compounds and were subjected to Argonaute immunopurifications, RNA was extracted and mRNA levels of the let-7 targets IGF2BP1 and HMGA2 were assayed by qPCR and represented as fold-change±s.e.m.. Representative experiment shown (n = 2). (TIFF) [file pone.0100951.s004.tiff]

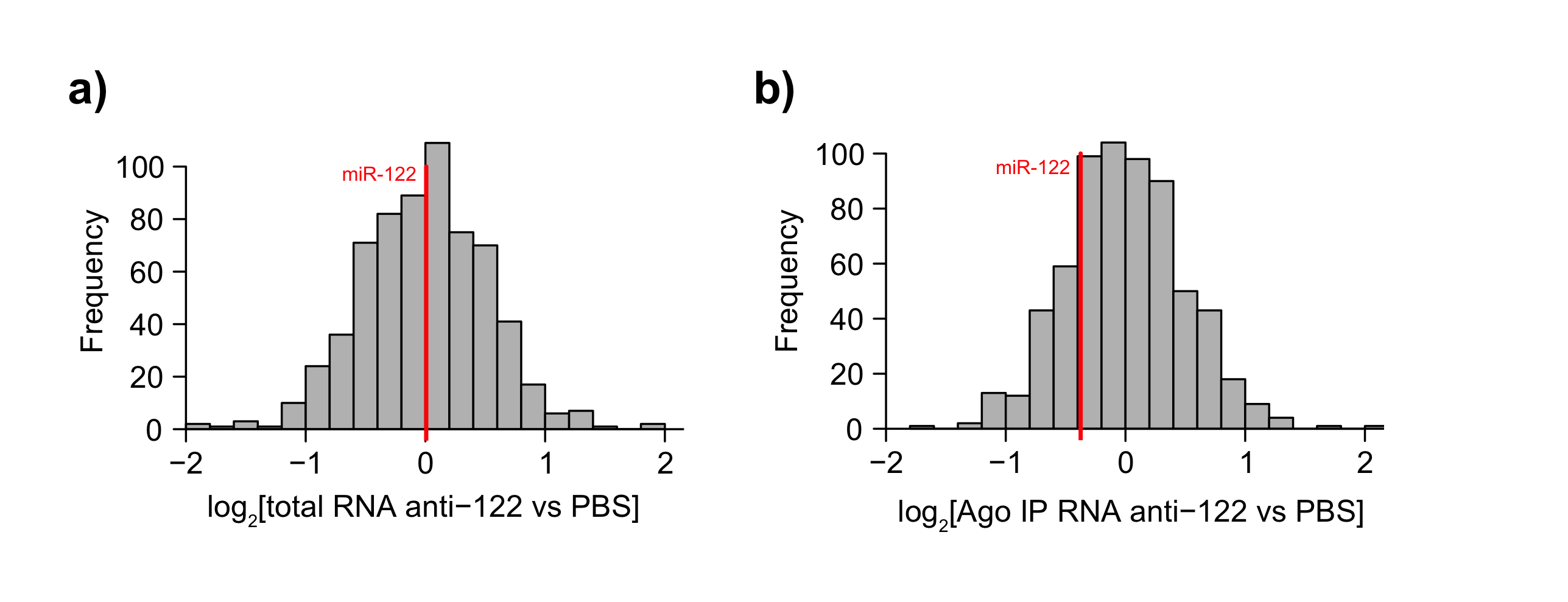

Supplement: Figure S5 — AntimiRs do not affect the levels of total mature or Argonaute-bound miR-122 in vivo . a) Histogram of the fold-changes for ∼200 miRNAs comparing anti-miR-122 treated livers vs PBS-treated livers following glyoxal removal of anti-miR. The log2 values across all miRNAs were mean centered at zero. miR-122 fold-change is highlighted by the red line (log2 = 0). b) Same as in (a) except for Argonaute IP RNA: miR-122 fold-change log2 = 0.77. Representative experiments shown from 2 independent experiments (n = 3). (TIFF) [file pone.0100951.s005.tiff]
